# Dental Organizations in the United States: Their Mistakes and Failures

**Published:** 1899-09

**Authors:** William H. Trueman

**Affiliations:** Philadelphia


### International Dental Journal.

Vol. XX. September, 1899. No. 9.

### Original Communications.[1]

### DENTAL ORGANIZATIONS IN THE UNITED STATES: THEIR MISTAKES AND FAILURES.[2]

BY WILLIAM H. TRUEMAN, D.D.S., PHILADELPHIA.

An editorial in the *Dental Digest*, April, 1899, page 284, entitled "Society Organization of the Dental Profession," in which the writer deplores the small representation of the dental profession actively engaged in society work, has suggested the following thoughts. It is but one of many lamentations which in various forms within recent years have appeared in our professional journals. They have been inspired by the too evident general indifference of the dental profession towards its professional organizations. Some have urgently appealed for an explanation of this indifference, its cause, and any suggestions which might tend to promote a more general interest in societies and society work. I do not recall any attempt to answer these. Very seldom, indeed, have I seen any plausible suggestion as to the cause or the remedy. On both these points the editorial in question simply suggests a change in meth-

---

[1] The editor and publishers are not responsible for the views of authors of papers published in this department, nor for any claim to novelty, or otherwise, that may be made by them. No papers will be received for this department that have appeared in any other journal published in the country.

[2] Read before the Pennsylvania Association of Dental Surgeons, June 13, 1899.

ods of selecting officers so as to have less politics, and to separate as much as possible business matters from scientific discussions. The real cause, and the real remedy, I am impressed, he has failed to touch.

When we reflect upon the very meagre results of sixty years organized effort to better the profession; that two national associations have during that time come and gone; that all over the country dental societies have been organized by the score; that they have been usually short lived; that, whether they have existed a few months or a score of years, they have passed out of existence leaving no visible assets, it is very evident that there has been a screw loose somewhere. Looking around, there is but little encouragement that this condition of affairs has materially changed. But one dental society in the United States, now active, has had a continued existence of half a century, and but few, if any, are doing anything more than to provide, in a hand-to-mouth fashion, for their present needs. The elements of permanency are wanting. A little quarrel, and the stronger party withdraws, organizes a new society, leaving their former comrades to struggle along for a little while and then to drop off and recruit the over-full ranks of the indifferent and the disgusted. It is not so in other professions. They have held together. They have looked beyond the present and have provided for the future. A society that spends part of its income in collecting and caring for a library and museum, or strives to possess some evidence of permanency, as have those of the medical profession in all of our larger cities, very soon has too much at stake to permit these little quarrels to take a serious turn. They have a home to love and care for; a common property, the value of which is a bond of union. It stimulates their pride, and its increase and care gives them something tangible to work for. The educational value of these libraries and museums is beyond compute. What encouragement is there for a dentist to invest in professional journals or in professional books, knowing, as he does, that when his short life is closed their usefulness is in all probability ended. To his executors they have no value, and are too often consigned to the paper-mill or the dump. He therefore buys as few as possible, and as there are no dental libraries to supplement his own meagre collection, his reading is restricted. His medical brother is far better provided. His professional society has made provision for his educational wants a prominent feature, and not only has he access to many large and well-arranged collections, but knowing that these medical libraries always welcome and properly care for anything left to them, he is encouraged to increase his own belongings, feeling, as he does so, that later on they will prove as useful to others as they have been to himself. He is not only broadened by his more extended reading, but loses more and more of self, is brought more in accord with his fellows, and feels, naturally, a keener interest in professional matters. His membership in a medical society having these advantages and privileges has a far greater value than has a membership in a dental society to his dental brother, conducted as most of them are.

I take this to be the fundamental error in dental society organizations in the United States. While, undoubtedly, they have had great educational value, and have done much, very much, for professional advancement, they have failed to reach, in this direction, possibilities. The stated meetings, the papers, the discussions, the social features, are all very good in their way; providing, as they do, means and opportunity for interchange of thought; they are, however, but passing events. We soon become familiar with the thoughts and ideas of our fellows in even a large society, and in a short time, with nothing else to depend upon, the proceedings lose interest and the best efforts become as an oft-told tale. Were these supplemented by opportunity for consulting a variety of professional journals and the educational advantages of a well-arranged library, a life and interest-giving variety would be introduced. Our views of men and things would be broadened, and the dental society become more useful and instructive. In order to bring this about we require a concentration of interests, fewer and stronger societies, more united as well as more active work. In my judgment, abandoning the older organizations and formation of new ones on slight provocation has been a serious mistake. They have had a history, and there is something very pleasing in keeping up the connecting links between the present and the past. We should have taken greater pride in supporting and maintaining those associations which in their day have done so much for our profession. We have been living too much in tents, having no settled home, no firm attachments; living in the present selfish lives, having no reverence for the past, no care for the future, and but little respect for any but ourselves.

It is this, I would suggest, which has largely hampered our professional advance. The only tangible evidence of solid growth is seen in the dental colleges. They have accumulated a little here and there from the past, and are to-day making this useful and adding to their inheritance. It is, however, in most cases, that which their poorly paid teachers have been willing to contribute to increase the usefulness of the institutions they served. What they gave, they gave to the colleges, not to the profession, and it is as truly private property as are the furnishings of your office or mine. Outside of this, notwithstanding that many members of our profession have acquired large fortunes, and dying have left a fair share of their accumulated wealth for the world's betterment, I cannot see that our profession, as a profession, is any richer to-day than it was sixty years ago. It has no open door inviting such benefactions, as other professions have; nor has it, to any appreciable extent, so far as I can see, provided for itself, added to, or in any way bettered its educational facilities. We have more dental journals. Only one, however, can be considered professional property. All the others are as much commercial enterprises as are the colleges. I mean no reflection on either in saying this. I simply desire to call attention to the fact that when we point to the dental colleges and the increase of dental literature of various kinds as an evidence of professional growth, it is well to bear in mind that they have been supplied by those who cater to our needs in much the same spirit as that in which our offices have been supplied with instruments and our laboratories with tools. That which members of the dental college faculties have contributed, which has remained with the colleges after they have left, is about all that has been unselfishly given, and but little of this, as I have before said, can be truly considered a professional belonging. I am well aware that from time to time attempts have been made to remedy this, but they have been poorly sustained. Of the many dental societies, I know of none which have reached a stage of assured permanency, none which have so far advanced in that direction as to warrant making them the recipient of a legacy with any assurance that it would prove of lasting usefulness. When we complain that the masses of the dental profession take but little interest in society work, let us ask, What have the societies to offer for their time, their interest, and their money? What can a dental society offer now to a prospective member more than it could sixty years ago? Is membership in a dental society more valuable now, or more to be desired, than it was then? In what respect? Why? Why not? These are questions which go to the gist of the whole matter.

With but few exceptions the dental societies of to-day offer to their members nothing more, and nothing better, than did those of more than half a century ago. The increase and the cheapening of periodic dental literature, and its consequent wider circulation and the facility it affords for interchange of thought, in some respects more pleasantly and more conveniently than at the dental society meeting, has to a far greater extent than is, perhaps, appreciated made the dental society as it now is less necessary to a progressive dentist. He may now, at his leisure, write his wants or contribute his mite, sure of finding an open door in some one of the many journals, and is there sure to meet a far larger and too often a more appreciative audience than the dental society affords. It is true, he may miss the verbal comments, the more rapid interchange of ideas possible by spoken than by written words, and the inspiring visible presence of auditor and audience; and quite as likely escape the rasping, temper-trying, ill-natured remarks of some dogmatic assertive boor. He will also escape the annoyance of having his labored and carefully prepared paper sandwiched between some trivial business affairs, or held until it becomes stale by a dilatory publication committee. The American Dental Association, so my experience there impressed me, was in these respects a sinner above all other sinners. Not infrequently unimportant and trifling business was needlessly injected between the reading of a paper and its discussion, or in the midst of a discussion, to so great an extent at times as to entirely break up the interest a well-prepared paper had incited. Members rising to speak in scientific discussions were curtly informed from the platform that they had no right to speak until they had paid their yearly dues. Again, visitors, known to be well informed upon matters before the Association, and on that account invited to speak, have been, as soon as they complied with the request, called down by an ill-mannered, discourteous member because, forsooth, the rigid enforcement of some petty rule, resolution, or by-law was, in his estimation, more important than a decent respect for a professional brother. More than this, its members have not only permitted, but they have repeatedly encouraged, by their applause and demonstrative enjoyment of the fun, silly jokes, violent aspersive language, the nonsense and antics of a circus clown, to break in upon serious professional discussions, or to be aimed at fellow-members, visitors, or those who had contributed papers or had taken part in the discussions. Is it any wonder that a society permitting this year after year should find its ablest men after a time becoming weary; or that the papers brought before it should show but little thought or care; or that the profession at large should take so little interest in it that finally, in the unclassic language of one of our able men, *"it petered out"?*

Both National Associations were extremely unfortunate. The first, on account of the very pronounced antagonism to amalgam on the part of its organizers, failed to secure, in the first place, that united support it otherwise would have received; and later, it failed to retain within its ranks many strong and progressive members of the profession. They claimed the right to practise as their experience and judgment suggested, and refused to assume, or to be bound by, any obligation that in the least degree curtailed this right. The lesser question was overshadowed by the greater. It was not, in reality, amalgam or no amalgam; it was much more than this. If the right of the society to interdict amalgam was conceded, why should it not take up other matters of practice and further hamper its members in their daily work. The local societies followed closely the lead of the National Association; and it is a significant fact that, of the many organized during this controversy, none that took any active part in it prospered. The same causes that operated so disastrously to the National Association more quickly, because of the more intimate relation of its members, ended the usefulness of these local societies.

There was thus spread widely, at the very beginning of organized effort for professional advancement, as the result of this attempted "bossism," a feeling of distrust and disgust, which through the unwise management of our societies, and the thoughtlessness or recklessness of those who have been permitted to poise as professional leaders, has been kept alive to the present day, and this has, undoubtedly, been an important factor in keeping away from the dental societies a large proportion of the members of the dental profession. The Mississippi Valley Association, although it adopted the amalgam resolutions, immediately forgot all about them and took no active part in the controversy, and lived to celebrate its golden anniversary. It ceased to exist only when changes of population made desirable a rearranging of society work. The Pennsylvania Association of Dental Surgeons, organized a little later, at a time when the battle waged hot, ignored the whole matter. It has from the very first stuck closely to business, and has confined its efforts exclusively to assisting its members in being helpful to each other, and advancing professional interests; it has never sought to hamper them in the slightest degree, and has always discouraged unprofitable controversy. Its meetings have been harmonious, and the work it has accomplished and the impress it has made upon professional matters few, if any, dental societies have equalled. It still lives, holding its regular meetings as it has been doing for more than fifty-two years, and is now the oldest dental society in the world. Had it not been for the unfortunate controversies which made united action and harmony impossible, the history of many of the early dental societies would have been equally satisfactory, and the relation of the dental profession to its professional societies would now be far more harmonious than it is. There is a lesson in this the thoughtful men in the dental societies would do well to heed. No dental society, either national or local, that courts controversy, or that is so organized or conducted as to encourage it, will ever prosper. The thinking men of our profession, the men who do the real work, and who alone are to be depended upon to give stamina and permanency to these organizations, must ever be reckoned with. They always have, and always will, insist upon the right to use, without constraint of any kind, their own judgment upon all matters of practice. This is, in their estimation, more valuable and more precious than is membership in a dental society. Whether the society insists upon a signed obligation, or the tacit acknowledgment of one by accepting membership, as did the older ones; or, under the plea of elevating its members to a higher standard,—as generally used, a senseless expression,—permits or encourages a hue and cry to be raised regarding matters of practice, the use of this or that remedy or device, or permits in discussion reflections upon the intelligence, skill, or integrity of its members, as both the National and many of the local societies have repeatedly done, the constraint is the same, and those subject to it, many of them, in the future will do as others have done in the past, leave the society rather than be subject to the taunts and jeers of their fellows, or abandon that which they have found satisfactory. In social intercourse, within very wide limits, we instinctively avoid encroaching upon the privacy of our associates' households, or attempting to dictate upon matters of purely personal taste. Why should we not exercise the same wise discretion regarding the professional relations of fellow-practitioners? What right have we to say what he shall use, or how he shall use it; what he shall do, or how he shall do it? Why should any one be accused of "abysmal ignorance," proclaimed a "slovenly fellow," a disgrace to his profession, or a mercenary knave, for speaking as his own studies and experience dictates, or using those methods which he has found most desirable and satisfactory to himself? Can we wonder that professional gentlemen, of which the dental profession is so largely composed, have given the "go by" to societies which have for so many years encouraged the use of these objectionable expressions, by so frequently permitting them in scientific and professional discussions? The dropping out of those who object to such things naturally left in the societies an undue proportion of those in whom gentlemanly instincts have been but imperfectly developed, and has compelled them to recruit largely from the same undesirable class.

The disgusted fathers have transmitted their grievances to their sons; the preceptors to their pupils; and thus it has come to pass that the profession has in this respect gone from bad to worse. Those who, despite of all this, while earnestly deploring it, have loyally held their place have been too few in numbers to check materially the growing evil. Here and there are local societies which are endeavoring by careful selection of members, by thoughtful and carefully planned organizations, and by example, to bring about a better state of things. It is, indeed, high time that it was done.

I would that we could stop here in reciting the causes which have brought about the condition of affairs the editorial referred to deplores. But the story would be incomplete. Our educational interests have furnished much cause for mischief. While admitting that years ago there was a marked laxity in dental educational institutions, I would remind you that this laxity was not by any means confined to them. It was common to all the professions in their early educational efforts. We must remember, also, that these dental colleges, which have been so freely and I think so unjustly censured, were, at the time that these changes could have been justly made, competing on very uneven terms with the private preceptor. It was very much easier, very much more convenient, and far less costly for a young man to spend a few days, weeks, or months, or even a year, with a village practitioner near his home, than to spend a year or two in a distant city. There was, indeed, but little encouragement for him to take the latter course, knowing, as he did, that on returning home he would likely find that a more economical although less ambitious competitor had in the mean time gathered up in his own intended bailiwick a fair share of the available material from which a practice is made. It was to the best interests of the profession that the colleges of that day made the prescribed course as easy and accessible as they did. Had they done otherwise it is quite likely so few would have accepted their terms that even to this day the private preceptor would have had by far the largest classes. It was only the wide distribution of college diplomas that made dental laws possible, and introduced this important factor in dental education. These laws have given to the colleges the support they needed to improve and advance. This they have steadily done. Naturally, as the dental enactments became more general and more exacting, and the private preceptor was thereby crowded out, the college classes increased in numbers. The profession in the public mind assumed greater importance, and as a means of livelihood has of late become vastly more popular. As these changes progressed, and in response to a demand, the dental colleges rapidly increased in number all over the country, with crowded classes, the commercial instincts of some of our professional brethren were aroused. They saw in all this nothing more than fewer or lesser fees, and became clamorous that something should be done to curtail this impending competition. They began to look upon the colleges as enemies, and naturally, impelled as they were by the same motives, adopted methods this thought suggested, borrowed from more humble callings.

While the advent of examining boards marks an important epoch in dental education, it has not proved an unmixed good. It was the first effective blow at the inefficient private preceptor. The examining board idea was introduced to prevent the entrance into the profession of the private preceptor's incompetent pupils, and to encourage students to seek the more thorough and systematic training the college course afforded. To this end, in the beginning, competent men were selected and empowered to examine all aspirants for dental practice unprovided with a college diploma. They were to judge whether their acquirements were or were not sufficient to warrant endorsement as dental surgeons. While there was at the bottom of all this the thought that those who had spent time and money to acquire a broad and thorough knowledge of the science should be legally protected from the competition of those who were, very many of them, grossly incompetent, nevertheless, there was, actuating those who organized the movement, a higher and nobler motive. These members of the dental profession, feeling keenly a responsibility for the irreparable injury constantly suffered by the community at the hands of incompetent practitioners, and a loss of prestige due to their many failures, felt justified in asking legal authority from the State to protect the public from damage and their calling from disgrace. Others of the profession, however, saw of the movement nothing more than its comercial side. It suggested to them a feasible method of restricting competition, and they hoped and expected that it would result in lessening the number admitted to professional rank. It did not, however, do so. Notwithstanding that the college course became practically obligatory, and that the dental colleges steadily raised their requirements, there has been a rapid increase in the number of dental students, calling for an increase in the number and capacity of the dental schools. Naturally this totally unlooked-for outcome of so promising a scheme proved to them a bitter disappointment.

As these matters developed, the discontented in our professional societies, urged thereto by reckless leaders who saw in this an opportunity of obtaining a prominent position in the profession with the smallest outlay of money, time, and labor, began in a thoughtless fashion to criticise the colleges. They had been persuaded that, somehow, the enactment of dental laws and their vigorous enforcement would bring them more business and larger fees. As neither were forthcoming, while the dental colleges were enjoying an undreamed-of prosperity, turning out graduates by the hundred, and brand-new signs appeared in close proximity to their own offices, they became clamorous for more actively aggressive measures. The colleges, as an open enemy, supplanted the private preceptor; as the dental examining board has suppressed the one, why should it not repress the other, and at once end this ruinous competition? That the competitor was in many cases the better man; that he in the natural order of things simply took the place of one who after many long years of efficient service was physically incapable of further holding the position in the community he had formerly honored, counted for nothing. It comes to all of us, if we live long enough, to know, as we look upon a youthful and vigorous competitor, that "he must increase, but I must decrease," yet how hard, how very hard, in many cases to gracefully acknowledge it. The dental examining boards had extirpated the pernicious private preceptor; why should they not be continued as a check upon the colleges? Equally, as when first organized, there was, outside and beyond commercial interests, a good purpose to be served in continuing the examining boards after the cause of their creation had practically ceased, *provided* that they were composed of wise and judicious men. This has not always been the case. Too often commercial interests have prevailed, and it has come to pass that men have been selected for that position simply because they were known as outspoken opponents of the colleges. Through this has crept in an evil that has done much mischief in our dental societies. For electioneering purposes, wild and malicious statements have been made concerning colleges and college teachers, worded in such a way that an answer was impossible. Unimportant matters have been magnified and twisted to the verge of positive untruths, and in too many cases the line has been overstepped in much the same spirit and with precisely the same motive that prompts labor leaders to foment trouble between employer and employed. Were these statements, so loudly made and so frequently repeated, at all definite, they could be at once disposed of, proved or disproved.

What can be done with such statements as these, taken from the May number of the *Dental Digest* (1899), page 363. They are found in an editorial in which it is contended that the so-called National Association of Dental Examiners are the body to fix the standards for the dental colleges. The writer claims that at least half of those who graduate never start in practice, and charges that this is all due to the colleges not weeding out those unfitted for the calling. As to how this is to be done he is discreetly silent. He further charges that the infirmaries in many schools are run to make money rather than to educate the students; that some colleges are controlled by the dental trust supply-houses, who insist that their students shall trade with them exclusively on pain of being "thrown" on examination day. These are sample statements that figure largely in discussions upon educational matters. They would be more to the point if they designated the colleges and the supply-houses implicated. It would be interesting and instructive to know, for instance, when asked regarding the relative merits of the various schools by young men about to begin a dental course, whether the editor refers to the Dental Department of the University of Pennsylvania as one of the colleges controlled by a dental supply-house; and also, what college or colleges the Dental Protective Supply Company has under *its* thumb. If true, what use is such indefinite information to me or to you as a guide to the standing of any one dental college in the United States? I characterize such statements, wherever and by whoever made, mean, cowardly, and malicious. They are prompted by no honest purpose. They arouse, and are intended to arouse, a feeling of distrust with no possibility of accomplishing the slightest good. (In making this reference I wish to state, in the most emphatic manner possible, that I have not selected the *Dental Digest* or the writer of its editorials as an object of criticism; for the journal and its editor I entertain feelings of the most profound respect. I have referred to it, and to him, in this connection from the simple yet curious fact that since planning this paper these matters have appeared in its editorial columns; their freshness and ready accessibility has prompted me to accept them in preference to others previously noted. They are "stock-in-trade" arguments, and common property.)

It would be, indeed, a glorious thing if the dean or the faculty of a dental college could, even by the end of the first year, with any chance of being just, say to a student, "I advise you to discontinue your studies. I see enough to know that you are simply wasting your time; you will never make a successful dentist." Can it be done? Please tell us how. Demonstrate its possibility before using the fact that all graduates do not turn out well as an argument to foment discord and distrust in our dental societies, or to impeach the honor and integrity of those in charge of our educational institutions. The initial education, social position, financial standing, important factors as they are at times, neither one nor all insure success, nor does their absence make it impossible. Experience has repeatedly proved this. The soothsayer's art is equally uncertain.

The evil effects of these loose irresponsible statements, which have for so long a time formed the staple detractive arguments in discussions of the dental educational question, especially in the State and National societies, are beyond measure. They pamper—professionally speaking—the vilest and most depraved tastes. Read, if you please, the articles published at various times and in a number of our dental journals by a member of the New Jersey State Dental Examining Board, sent broadcast as specimens of the educational standing of graduates from American Dental Colleges,[1] containing a number of answers culled from examination papers submitted to the board. I know he states that they are not all as bad as those he gives, but what good purpose was served by making the exhibit at all? What other intention could the board have, or could it have had, in permitting their publication than to magnify their office and belie the colleges. Some of those answers, to my

---

[1] Items of Interest, vol. xix., August, 1897, page 553.

notion, were faulty only in spelling and grammar, and, considering the circumstances under which they were written, were far more excusable than is the bad taste and breach of trust in making them public. No more are they a reflection upon the educational status of graduates from American dental colleges than is the article from the same source found in the August number of the INTERNATIONAL DENTAL JOURNAL, 1898, page 558, a reflection upon the profession of which he is a member. It simply shows that professional ethics in the dental profession must be at a low ebb in the State of New Jersey to permit such things.

The most rigid and exacting rules do not prevent, as our police reports too frequently show, dishonest men obtaining employment in well-managed banks and business houses; even the safeguards around the sacred desk, at times, are inefficient in preventing wolves in sheep's clothing playing the *rôle* of religious teachers. Now and again colleges graduate men they ought not; likewise dental societies sometimes admit as members, even place in responsible positions, men who do them no credit.

The liberty allowed in our dental societies in these cowardly attacks upon the teachers and the colleges has been most demoralizing. If a college is doing wrong, or a teacher in it acting unprofessionally, and a member feels that it is his duty to make it known, let him do so openly like a man, giving the names with the facts. If this had always been insisted upon, how quickly these discussions would have been brought to an end. How much less friction we would have had. How much more unity and harmony; how much more respect for our colleges, our teachers, our societies, our profession, and ourselves.

We lament that so few young graduates become interested in society work. How can they? Is it not natural that a young man should hold aloof from a society that permits, that encourages its members to belittle the college whose diploma he holds? Can you expect him to affiliate with men who in season and out of season assert that the teachers he has learned to honor and respect cheat half the students who come under their care, taking from them their money and giving nothing valuable in return? How does he know but that you refer to him, and his alma mater? He does not ask, he does not care. If he has within him the making of a true, manly, professional gentleman, his whole soul revolts at the thought of uniting with such a set of character assassins. The atmosphere of such a dental society is so different from that of the college that it stifles him; he prefers to be outside; and let me say, *I honor him for his choice.*

I am one with those who desire a higher and broader educational standard for our profession, but see no reason why we should wait until a new generation has grown up to obtain it. Let it begin right in our dental societies, and begin *now*. The members of the societies, many of them, need it far more than do the students. First of all, let us learn to treat each other as gentlemen; enforce in all the societies, from the National down, that strict discipline recognized in all clubs of well-bred, self-respecting gentlemen. Send the backbiters, those who are aping the Debs and Irons of trade-union fame, to coventry, and *make them stay there.* We want, most of all, harmony and united action between the societies and the educational institutions. We need on the examining boards broadminded, dignified professional gentlemen, who have the good of the profession at heart, men of character and standing, who can command and retain the respect of their fellows. We need such men as these most of all, and most urgently in the National body, to replace the men who have so signally proved their unfitness for the position they hold. We must have harmony, and to secure it can best spare the men who in the past have ever been promoters of strife. If we would advance along professional lines, we must rise above that commercial spirit which sees in each addition to our ranks a business competitor, and strive to help each other to a higher, nobler, better professional life. Let us make our dental societies post-graduate colleges; providing within them the means by which each and all may find something suited to their needs; make them societies that it is worth while to belong to. Let us learn to down that selfish spirit which sees nothing good in what another does, feels no interest in another's welfare, and takes no pride in our profession's future. When we fraternize, let us be as David and Jonathan, not like Cain, who slew his brother.

